# The Impact of Fermentation on Bee Pollen Polyphenolic Compounds Composition

**DOI:** 10.3390/antiox11040645

**Published:** 2022-03-28

**Authors:** Vaida Adaškevičiūtė, Vilma Kaškonienė, Karolina Barčauskaitė, Paulius Kaškonas, Audrius Maruška

**Affiliations:** 1 Instrumental Analysis Open Access Centre, Faculty of Natural Sciences, Vytautas Magnus University, LT-44404 Kaunas, Lithuania; vaida.adaskeviciute@vdu.lt (V.A.); audrius.maruska@vdu.lt (A.M.); 2 Lithuanian Research Centre for Agriculture and Forestry, LT-58344 Kėdainiai District, Lithuania; karolina.barcauskaite@lammc.lt; 3 Institute of Metrology, Kaunas University of Technology, LT-51368 Kaunas, Lithuania; paulius.kaskonas@ktu.lt

**Keywords:** bee pollen, solid-state fermentation, UPLC, antioxidant activity, flavonoids, *Lactobacillus rhamnosus*, lactic acid

## Abstract

Bee-collected pollen is one of the most valuable natural products. However, the pollen cell walls limit the digestibility and release of nutrients to the human body. Solid-state lactic acid fermentation can be used to ease the release of bioactive compounds from the pollen cell. The aim of this research was to determine the impact of a solid-state lactic acid fermentation process on biologically active compound composition and antioxidant activity of bee-collected pollen from various European regions (Italy, Netherlands, Lithuania, Poland, Sweden, Denmark, Malta, Slovakia, and Spain). Spontaneous fermentation and fermentation using an *L. rhamnosus* culture were performed. The total content of phenolic compounds, total content of flavonoids, and radical (DPPH) scavenging activity were measured by spectrophotometric tests, while UPLC was employed for quantification of phenolic compounds. The determined fermentation positive effects included an increase of total phenolic content by 1.4–2.3 times, total flavonoid content by 1.1–1.6 times, and radical scavenging activity by 1.4–2.3 times. Naringenin (21.09–135.03 µg/g), quercetin (6.62–78.86 µg/g), luteolin (29.41–88.90 µg/g), and rutin (21.40–89.93 µg/g) were the most abundant flavonoids in all samples; however, their variation level was both geographical in origin and fermentation-type dependent. Fermentation increased the content of phenolic acids with high antioxidant potentials such as ellagic, ferulic and caffeic, while reduction of chlorogenic acid was determined.

## 1. Introduction

Nowadays, it is widely accepted that diet and a well-balanced lifestyle play an increasing role in the prevention of disease. The perception of food production and consumption is currently changing, and the development of functional food is an important part of the food market. In order to improve quality of life, modern consumers are increasingly concerned about using natural functional foods containing biologically active substances from natural sources, partly because they have better safety than synthetic drugs [1]. Scientific research describes bee products as having many benefits for health, including antibacterial, anti-atherosclerotic, anti-inflammatory, anti-cancerogenic, hepatoprotective, antiviral and antioxidants properties [2].

Bee-collected pollen, a natural constituent of flowers, which determines the ability of plants to reproduce, is in the form of granules and can be stored by bees for food supplies [2]. A variety of biochemical compounds such as carbohydrates, vitamins, enzymes, fatty acids, polyphenols, lignans, bioactive peptides, minerals, probiotics has been found in bee pollen chemical composition. These compounds have significant therapeutic and preventive potential with respect to a weakened immune system, as well as arteriosclerotic, cardiovascular, neurodegenerative and cancer diseases [3,4].

Pollen cells are covered by a double layer consisting of an outer exine layer protecting the reproductive cells from environmental influences and an inner intine layer surrounding the protoplasm of the pollen grain. This cell wall structure leads to poor digestibility and release of valuable pollen compounds; consequently, biologically useful compounds and nutrients are not assimilated [2]. In order to improve the absorption of these valuable natural substances in the simplest possible way, it is necessary to find, evaluate and apply various bee-collected pollen treatment methods such as fermentation, enzymatic hydrolysis or other physical methods.

The aim of this research was to determine the impact of a solid-state lactic acid fermentation process on biologically active compound composition and antioxidant activity of bee-collected pollen from various regions of Europe. Obtained data will help characterize the possible methods allowing increased digestibility and release of biologically valuable pollen compounds to the human organism. To our knowledge, this is the first study revealing detailed changes in phenolic compound composition of bee-collected pollen after lactic acid fermentation. It is important to note that the bee pollen fermentation process is sensitive not only to the selection of appropriate microorganisms (lactic acid bacteria and/or yeast), and the determination of the optimal temperature and duration of the process, but also to the phytochemical composition of pollen resulting from its botanical origin which, in turn, strongly relates to the geographic origin of the pollen.

## 2. Materials and Methods

### 2.1. Pollen Samples

Nine bee-collected pollen samples from various Europe regions investigated in this research are listed in Table 1. Pollen was collected during flowering season from May to August in 2018. The samples were retained in a refrigerator at +5 °C for a maximum of four weeks. They were homogenized with a pestle and porcelain mortar before the analysis and extract preparation procedures.

### 2.2. Chemicals and Reagents

Hexamethylenetetramine (≥99%), lactic acid (≥98%) and aluminum chloride (98%) were obtained from Carl Roth Gmbh & Co Kg (Karlsruhe, Germany). 2,2-diphenyl-1-picrylhydrazyl (DPPH) (99%), chlorogenic acid (≥95%), ferulic acid (≥99%), salicylic acid (≥99%), methanol (≥99.9%), hesperidin (≥90%), benzoic acid (≥97%), myricetin (≥96%), rutin (95%), naringenin (≥95%), coumarin (≥99%), caffeic acid (≥98%), vanillic acid (≥97%), gallic acid (≥98%), ellagic acid (≥97%), and syringic acid (≥97%), of analytical grade were obtained from Sigma-Aldrich Corp (Taufkirchen, Germany). Folin-Ciocalteu reagent, trifluoracetic acid (99%) and acetonitrile (≥99.8%) were supplied by Merck KGAA (Darmstadt, Germany). MRS with Tween 80 broth was obtained from Biolife Italiana S. r. l. (Milan, Italy). Coumaric acid and luteolin were obtained from: Chromadex, Inc. (Los Angeles, CA, USA). Sodium carbonate and acetic acid (99.9%) were bought from Reachem S.r. o. (Bratislava, Slovakia). Bidistilled water was prepared by means of distillation apparatus Thermo Fisher Scientific, Inc. (Fremont, CA, USA).

### 2.3. Solid State Fermentation Bacteria

For solid-state fermentation an inoculum of *Lactobacillus rhamnosus* GG (ATCC 53103) (Gefilus, Valio Ltd., Helsinki, Finland) was used. The viability of the bacterial culture was restored in MRS broth with Tween 80, and plates with bacterial culture were prepared according to Kaškonienė et al. [5]. Bacterial fermentation using *L. rhamnosus* and spontaneous fermentation, i.e., without addition of bacteria, were conducted according to the method described by Kaškonienė et al. [6] with small modifications. The samples for bioprocessing were prepared in 10 mL vials. Briefly, 10 g of each pollen sample were moistened with 2 mL of sterile distilled water for 2 h then heated and cooled. A mixture of multifloral spring honey collected in Lithuania together with water (1.5 g of honey with 2.5 mL of water) was added. Subsequently, 800 µL of *L. rhamnosus* (2.9 × 10⁹ colony-forming units (CFU)/mL) for bacterial fermentation, and 800 µL of MRS broth with Tween 80 for spontaneous fermentation, were supplemented. Vials with about 20% of space above the pollen were sealed and placed into an incubator (Biosan, Ltd., Riga, Latvia) for fermentation at +37 °C. The optimal duration of the fermentation, determined and described by Adaškevičiūtė et al. [7], was used for the process of the prepared samples: 9 days for bacterial and 11 days for spontaneous fermentation.

### 2.4. Preparation of Extracts

Two grams of pollen samples were extracted with 20 mL 80% methanol before and after fermentation. Samples to extract before fermentation (natural pollen samples) were prepared in the same manner as samples for spontaneous fermentation to exclude variation of the results because of the additional water, MRS broth and honey; the extracts were prepared straight after mixing of all components.

After 24 h of extraction at room temperature, samples were filtered through 7–10 μm paper filter and repeatedly filtered using a 0.22 μm polyvinylidene fluoride (PVDF) membrane filter (BGB Analytik USA LLC, Alexandria, VA, USA) for all spectrophotometric tests and UPLC analysis [5].

### 2.5. Spectrophotometric Evaluation

Total phenolic compound content (TPC), total flavonoid content (TFC) and radical scavenging activity (RSA) were determined according to spectrophotometric methods described in Kaškonienė et al. [8] with small modifications for adaption to a microplate reader. TPC was evaluated using the Folin-Ciocalteu method. TFC was determined using AlCl_3_ colorimetric method and RSA was obtained using the 2,2-diphenyl-1-picrylhydrazyl (DPPH) free radical colorimetric reaction method. Measurements were performed with a Hipo MPP-96 spectrophotometer (Biosan, Ltd., Riga, Latvia). Rutin calibration curves were prepared for each reaction. Results are expressed as mg of rutin equivalent (RUE) per 1 g of prepared sample. A summary of used methods, according to Adaškevičiūtė et al. [9], is presented in Table 2. 

### 2.6. UPLC-DAD Evaluation

Quantitative and qualitative analysis of natural and fermented pollen samples were performed using UPLC-DAD. Chromatography was carried out with an Acquity UPLC H-Class CM Core System chromatograph with a Diode-Array Detection (DAD) system (Waters Corp, Milford, CT, USA). The DAD was set for compound identification from the 210 to 400 nm wavelength region using a three-dimensional scan mode. The maximum absorbance of the corresponding compounds was provided at 280 and 305 nm.

Validation of the method was performed with standard solutions of ferulic, benzoic acid and quercetin to prove the specificity, accuracy, repeatability, and linearity of the method. A specificity parameter was evaluated comparing chromatograms of a mixture of standard solutions and the individual standard solutions and retention times of the obtained peaks. Repeatability was evaluated using five injections of each standard. During method accuracy evaluation, standard solutions were injected within five days. Observed standard deviations of the retention times during these tests did not exceed 5.00%. Linearity was evaluated using six point injections of each standard. Regression coefficients of calibration curves met the R² ≥ 0.99 requirement (Table 3).

Pollen sample extracts were diluted six times with 80% methanol before UPLC analysis. The separation of phenolic acids and flavonoids in 5.0 µL of injected extract was performed using a 1.7 µm particle-based reverse-phase column BEH C18 (Waters Corp, Milford, CT, USA) of 150 mm length and 2.1 mm inner diameter. The mobile phases consisted of two solvents: phase A based on water with 0.1% TFA, and phase B based on acetonitrile with 0.1% TFA. The 15.0 min gradient program was set as follows (%B): 0–1.0 min 1%, 1.0–10.0 min 30%, 10.0–12.0 min 95%, 12.0–12.1 min 1%, 12.1–15.0 min 1%. The flow rate was set at 0.65 mL/min and column temperature was set at +45 °C [10].

Chromatograms were recorded and data were obtained using MassLynx 4.0 software (Waters, Milford, CT, USA). Qualitative analysis was performed comparing the retention time of bee-collected pollen compounds and standard substances. The number of phenolic compounds was calculated according to linear regression equations of the calibration curves prepared with each standard in the range 0.01–0.50 mg/mL. The results of qualitative analysis are expressed in µg of compound per 1 g of sample.

### 2.7. Statistical Analysis

All spectrophotometric measurements of each pollen type were performed 10 times, or with UPLC six times. Data systematization was performed using MS Excel 15.11.2 (2015, Microsoft Corp, Redmond, Washington, USA) software. Chemometric analysis of the results was done using MATLAB v9.1.0 (R2016b, The MathWorks, Inc., Natick, MA, USA) software.

The data set representing tested pollen samples was composed of 27 tested cases (nine samples before fermentation = natural samples; nine samples after spontaneous fermentation, and nine samples after bacterial fermentation), while each of them was described by three variables (TPC, TFC and RSA).

Data preprocessing involved a standardization procedure carried out by subtracting the mean of the variable and dividing by its standard deviation. Statistical analysis included analysis of variance (ANOVA), correlation analysis and hierarchical clustering analysis (HCA) [9,11,12]. ANOVA was applied for hypothesis testing to find statistically significant changes in measured TPC, TFC and RSA values after fermentations at the selected significance level *p* ≤ 0.05. HCA was employed to compare the closeness of samples according to an applied similarity measure (Euclidean distance) and to reveal how fermentation affects grouping tendencies of the tested samples. To evaluate the relationship between the measured TPC, TFC and RSA (pair-wise), Pearson’s linear correlation coefficient was evaluated at the statistical significance *p* ≤ 0.05.

## 3. Results and Discussion

### 3.1. Variation of Total Phenolic Compounds, Flavonoid Content, and Radical Scavenging Activity in Fermented and Non-Fermented Bee-Collected Pollen

Nine samples of bee-collected pollen were prepared and fermented using *L. rhamnosus* and spontaneously. Spontaneous fermentation occurs without addition of the bacteria because of the native pollen microflora. All samples were assessed by comparing the chemical composition, TPC, TFC and RSA measured before and after fermentation process. The observed variations of TPC, TFC and RSA after fermentation of the tested samples were statistically significant at *p* ≤ 0.05. The results are presented in Table 4.

Fermentation significantly (*p* ≤ 0.05) increased TPC from 12.0 up to 89.1%. The level of the changes was depended on fermentation type (bacterial or spontaneous), and geographical origin of the pollen, which in turn may be strongly related with botanical pollen origin. Variations of the pollen samples’ collection time and visual color difference (see Figure S1) supported the assumption that the botanical origin and chemical composition of the samples were different (however, the evaluation of the botanical composition of the pollen was not within the scope of this study). The greatest content of TPC both before fermentation and after bacterial/spontaneous fermentations was determined in Lithuanian pollen, while the lowest content was found in pollen from Malta. Yan et al. [13] studied various types of pollen fermentation and determined that this bioprocess increases the total amount of phenolic compounds by 17.8%. The group of microorganisms providing the highest amounts of biologically active compounds during fermentation was also assessed in the study [13]. According to the authors, in order to degrade the pollen cell wall as much as possible and thus obtain the highest possible amounts of TPC, it is advisable to carry out the fermentation with a mixture of yeast and lactic acid bacteria. In our research an observed increase of TPC after bacterial *L. rhamnosus* fermentation ranged from 23.9 to 89.1%. In comparison with the mentioned study from China, the differences of the results could be explained by the species and strains of microorganisms used in the bioprocess, which have different properties and effects on pollen and pollen itself.

All bee-collected pollen had a similar amount of TPC before fermentation (8.08–11.97 mg/g (RUE)), but after bacterial/spontaneous fermentation the amounts increased. The increment was dependent on geographical origin of the samples. These results can be explained by the fact that the geographical location is closely linked to the climate. It can be assumed that in colder climate areas pollen tends to accumulate higher amounts of biologically active substances, and therefore higher amounts are released during fermentation. Pollen from the northern part of Europe had significantly higher amounts (1.2–1.9 times) of biologically active substances after fermentation than pollen from the south (*p* ≤ 0.05).

Bee-collected pollen fermentation is studied more in South American countries, and a small number of such studies have been conducted in European countries. The TPC increased from 13.34 ± 3.61 to 18.89 ± 2.24 mg/g (expressed as gallic acid equivalents) after fermentation of Columbian bee-collected pollen with lactic acid bacteria [14]. The study also showed the importance of the geographical origin of pollen: after bacterial fermentation TPC in the discussed study increased 1.4 times, while in our study it increased 1.9 times. Therefore, it could be assumed that the colder climate zones result in a tendency of pollen to accumulate higher amounts of biologically active substances.

TFC in tested pollen samples before and after fermentation significantly increased by 1.1–1.6 times at the significance level *p* ≤ 0.05 (Table 4). The greatest amount of TFC was determined in a pollen sample from Lithuania (6.26 ± 0.11 mg/g (RUE), 9.67 ± 0.13 mg/g (RUE) and 8.84 ± 0.18 mg/g (RUE) before fermentation, after fermentation with lactic acid bacteria and after spontaneous fermentation, respectively). The lowest TFC amount was found in a Maltese bee pollen sample (3.69 ± 0.11 mg/g (RUE), 5.41 ± 0.05 mg/g (RUE) and 4.99 ± 0.09 mg/g (RUE), respectively).

TFC is closely related to the TPC, thus the observed trends are quite similar. Strong correlations between TPC and TFC before fermentation and after bacterial/spontaneous fermentation were determined (0.905, 0.979 and 0.906, respectively). However, as was expected, the values of TFC were remarkably lower than TPC. The difference might be explained by the fact that the Folin-Ciocalteu reagent reacts not only with flavonoids, but also with phenolic acids and other reducing agents such as gallic, caffeic and chlorogenic acids, which were detected in the pollen (see Section 3.2). The total flavonoid method is more selective, and is able to detect flavonoids containing flavone and flavonol groups. Therefore, the determined TPC content was higher than TFC.

An increase of TFC was also determined in research by Kaškonienė et al. [6], where flavonoid content changed from 1.6 to 2.4 times after fermentation. The same tendency of the TFC was also observed after spontaneous/bacterial fermentation in the study of Latvian pollen: after spontaneous fermentation, concentration changed 1.6–2.1 times, after fermentation with *L. lact* is 1.7–2.2 times, and after fermentation with *L. rhamnosus* 1.8–2.4 times [5]. The results of TFC published by other authors resemble TFC values obtained in our study.

The increment of evaluated RSA after spontaneous/bacterial fermentation was statistically significant and ranged from 35.3% to 133.5% at *p* ≤ 0.05 (Table 4). An increase of RSA was also determined in other studies. In a study of Indian pollen, the activity changed from 67% (before fermentation) to 86% (after fermentation) using *L. lactis* culture for the bioprocess [14]. In a study of Latvian pollen, RSA increased 1.3–1.9 times after fermentation with *L. lactis* bacterial culture, 1.5–2.0 times after fermentation with *L. rhamnosus* and 1.4–1.7 times after spontaneous fermentation [5]. Slightly different results were obtained in our study, with a1.6–2.3 times increase after fermentation with *L. rhamnosus*, and 1.4–2.1 times increase after spontaneous fermentation. It is evident that microorganisms used for fermentation and botanical origin of the pollen have a high impact on the fermentation yield. Although the bioprocess of different botanical origin pollen was carried out with different cultures of microorganisms in the observed studies, a clear benefit of fermentation was found relative to antioxidant properties and biologically active substance content.

The difference between TPC and RSA values can be explained by the different structure of flavonoids and phenolic acids identified in the pollen. In the scientific literature, three criteria, known as Bors criteria, have been proposed for explanation of the antioxidant activity of phenolic compounds. These criteria are a catechol group in the B-ring, a 2,3-double bond in conjugation with the 4-oxo group, and 3 and 5-hydrohyl groups in combination with the 4-oxo group [15]. Platzer et al. compared antioxidant activity of different phenolic compounds (hydroxycinnamic and hydroxybenzoic acids, kaempherol, hesperetin, (+)catechin, etc.) using Folin Ciocalteu and DPPH assays [16]. It was concluded that antioxidant activity is strongly related with the structure of compounds including the number of hydroxyl groups and agreement to Bors criteria. Furthermore, it should be noted that all reference compounds showed responses in the Folin Ciocalteu assay, but not all to DPPH, especially flavanones and dihydrochalcones [16]. Flavanones naringenin and hesperidin were identified in our study, while the first was one of the major compounds detected in pollen (see Section 3.2). That could explain lower RSA values than TPC. However, contrary to the study with reference compounds [16], a strong correlation between TPC and RSA was determined. Correlation coefficients were in the range between 0.892 and 0.945 depending on fermentation type (see Section 3.3).

Zuluaga-Dominguez and Quicazan [17] investigated the fermentation of pollen and found that the use of a mixture of lactic acid bacteria and yeast in the bioprocess increased antioxidant activity by 30–39%. The study also showed that the fermentation of pollen with a single culture—with lactic acid bacteria or with yeast—reduces the antioxidant activity. Disagreement of these results with our study may be because of the type of microorganisms used. The lactic acid bacteria *L*. *plantarum*, used in the Zuluaga-Dominguez and Quicazan [17] research, seems to use amounts of biologically active substances in the natural raw material for their own needs, therefore observed RSA did not increase after fermentation. The *L*. *plantarum* metabolic processes during fermentation was also described in Munoz et al. [18]. According to the authors, this lactic acid bacterial culture used in fermentation of pollen decomposes only certain phenolic acids, such as gallic, hydroxycinnamic acid, catechol or methyl gallate.

### 3.2. Qualitative and Qualitative Analysis of Phenolic Compounds and Lactic Acid

Analysis of biologically active substances by ultra-performance liquid chromatography with a diode array detector (UPLC-DAD) was performed to evaluate the influence of fermentation on the qualitative and quantitative composition of phenolic compounds in pollen samples. During the study, validation of the method was performed, and all parameters met requirements. Accuracy and repeatability standard deviations were 0.01% and linearity R² = 0.999 of each standard listed in Table 3. Seventeen standard solutions were used to identify the predominant phenolic compounds in the samples, which were evaluated both before and after bacterial/spontaneous fermentation. UPLC-DAD also allowed evaluation of the content of lactic acid, which is an important parameter of successful lactic acid fermentation and helps to preserve bee bread in the hive [1,19].

**Figure 1:**
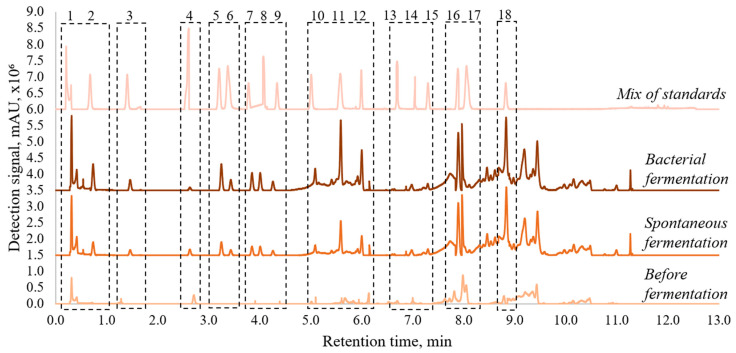
Chromatographic profiles of Lithuanian pollen sample (1—lactic acid, 2—gallic acid, 3—benzoic acid, 4—chlorogenic acid, 5—vanillic acid, 6—caffeic acid, 7—syringic acid, 8—salicylic acid, 9—coumaric acid, 10—ferulic acid, 11—rutin, 12—ellagic acid, 13—coumarin, 14—myricetin, 15—hesperidin, 16—quercetin, 17—luteolin, 18—naringenin).

The analysis revealed the dependence of the profiles of tested pollen extracts on geographical origin and the fermentation process. Figure 1 represents a visual example of the changes in composition profile after fermentation of a Lithuanian pollen sample. Detailed results of phenolic acids and flavonoids are presented in Table 5.

The different geographical origin of pollen is responsible for qualitative differences in biologically active substances. It was determined that according to profiles, three groups of samples can be distinguished: Danish, Dutch and Swedish pollen samples; Slovak, Polish, Lithuanian and Maltese pollen samples; Italian and Spanish pollen samples. The greatest diversity of biologically active compounds was established in Lithuanian, Slovak and Polish pollen, while the lowest was in Spanish, Italian and Maltese samples. Ferulic, ellagic acids, rutin, quercetin, luteolin, and naringenin were identified in all tested samples. Lactic acid, as the main product of fermentation, was identified in all samples. These beneficial micronutrients may have therapeutic potentials in of bee pollen. Rutin, quercetin, ferulic and ellagic acids were also identified in natural pollen extracts by other researchers [20,21].

The results of the qualitative analysis of pollen extracts by the UPLC-DAD method reflect not only the main compositional similarities, but also the differences between the samples of different origins. For example, gallic acid was not identified in Italian pollen, benzoic acid in samples from Malta, caffeic acid in Swedish, Slovak and Spanish samples, salicylic acid in Dutch and Italian pollen, and coumaric acid in Swedish, Polish and pollen from Malta. On the other hand, coumarin was identified only in Danish, Swedish and Italian pollen, and hesperidin was determined only in Lithuanian, Dutch and Maltese pollen samples. Interesting is that, according to the literature, rutin is the most detected glycoside in bee-collected pollen and it is the main component of bee bread [22]. Consequently, an increase of rutin peak intensity is noticeable after fermentation of pollen samples. Caffeic, chlorogenic, ferulic, coumaric, and gallic acids, as well as quercetin and naringenin, were determined in pollen and bee bread in other studies [23,24]. These investigations showed that after fermentation of pollen, metabolic activity determines the formation of similar components to those in bee bread.

Qualitative analysis showed that fermentation influenced the variety of biologically active substances. The intensity of most of the peaks in chromatograms after fermentation also were much higher than before fermentation (Figure 1). For example, in Lithuanian sample vanillic and caffeic acids were identified only after spontaneous or bacterial fermentation (Table 5). Danish pollen revealed vanillic and benzoic acids, and hesperidin after bioprocess; Swedish, gallic, benzoic, vanillic, syringic acids and myricetin; Polish, benzoic and vanillic acids, and myricetin; and Slovak, Maltese, Dutch, benzoic and vanillic acids, and hesperidin. According to the literature, the formation of phenolic acids is affected by lactic acid bacteria’s ability to decompose certain compounds to benzoic and vanillic acids. These acids ensure a longer shelf life and help to protect products from spoilage, as these substances stop the development of both pathogenic bacteria and fungi [25,26,27].

Assessing the influence of fermentation on the chemical composition of pollen samples from various Europe regions, it was found that the amounts of the most analyzed compounds increased by 1.2–3.1 times after spontaneous/bacterial fermentation (*p* ≤ 0.05). However, the loss of compounds can also occur during fermentation. A decrease of up to 49.5% of chlorogenic acid was observed in this study. The degradation of chlorogenic acid was especially expressed after bacterial fermentation, which can be caused by the activity of lactic acid bacteria. According to the published data, lactic acid bacteria hydrolyze chlorogenic acid using esterases, that results in a decrease of the amount of this acid in the pollen samples [28,29].

Naringenin (21.09 ± 0.21–135.03 ± 0.49 µg/g), quercetin (6.62 ± 0.41–78.86 ± 0.46 µg/g), luteolin (29.41 ± 0.27–88.90 ± 0.46 µg/g) and rutin (21.40 ± 0.21–89.93 ± 0.53 µg/g) were predominant in all samples. The content was depended on fermentation type and origin of the sample. Lithuanian and Slovak pollen had the highest amounts of phenolic acids and flavonoids, while Italian and Maltese pollen had the lowest values.

Literature data is scarce concerning quantitative composition of phenolic acids of pollen collected in various regions of Europe. Sawicki et al. [30] did not detect quercetin (while it was detected in all our studied samples) or caffeic acid (it was detected in five out of nine samples in our study) in bee pollen. However, the variation in polyphenol composition proves that bee pollen is very complex from a chemical composition point of view. Rutin (2.00–53.41 µg/g) and salicylic acid (1.19–25.70 µg/g) were the major compounds in the fresh bee-collected pollen samples from Poland collected over several years [18], while chrysin (0.07–0.08 µg/g), kaempferol (0.05–0.24 µg/g), and apigenin (0.03–0.23 µg/g) were minor compounds in the same study of Polish pollen. Comparing these data with our results, a quite similar content of salicylic acid and rutin was detected in pollen from Poland. Nevertheless, in [21], high variability of both component concentrations was identified. This difference in the results may be due to the sample state (fresh or dried), extraction method and the solvent used. Scientific publications suggest that organic solvents disrupt cell walls and are better solvents for the extraction of phenolic acids from plant raw materials, so it can be assumed that this factor affected rutin and salicylic acid obtained in Polish and Korean studies [21,31]. Furthermore, different geographical region and the botanical origin of pollen could influence both quantitative and qualitative phenolic compound composition in samples from the same country.

As was mentioned earlier, lactic acid preserves bee bread after natural fermentation in a beehive [1]. Changes in lactic acid were determined in our study. The content of lactic acid varied depending on the geographical origin of the pollen and on fermentation type: 14.13 ± 0.41–59.24 ± 0.53 µg/g were determined before fermentation; 18.34 ± 0.41–73.33 ± 0.53 µg/g after bacterial fermentation, and 15.61 ± 0.30–69.63 ± 0.12 µg/g after spontaneous fermentation. The lowest amount of lactic acid was detected in pollen collected in Italy, Spain and Malta (14.13–18.32 µg/g, 18.34–23.02 µg/g and 16.61–22.0 µg/g before fermentation, after bacterial fermentation, and after spontaneous fermentation, respectively). The results suggest that pollen in these countries accumulates lower amounts of carbohydrates. The content of carbohydrates may vary from 24–60% [1]. After bacterial fermentation, lactic acid content significantly (*p* ≤ 0.05) increased by 1.2–2.0 times, while after spontaneous fermentation a significant (*p* ≤ 0.05) increase by 1.1–1.5 times was determined in seven samples out of nine. According to the literature, the content of lactic acid in bee bread is six times higher than in pollen [1].

### 3.3. Chemometric Analysis of the Samples

**Figure 2:**
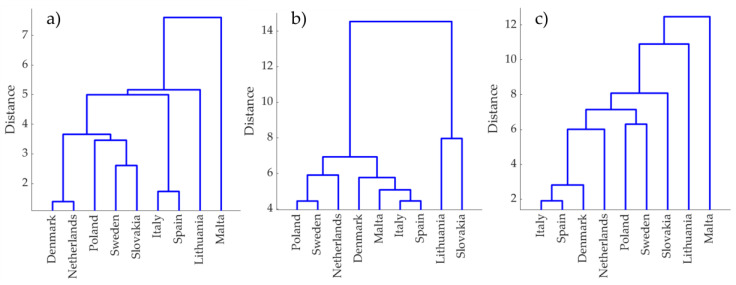
Hierarchical cluster analysis dendrograms according to evaluated TPC, TFC and RSA: (**a**) before fermentation, (**b**) after spontaneous fermentation, (**c**) after bacterial fermentation.

Three dendrograms were built using HCA to reveal similarities of the pollen samples from different geographical origin according to measured TPC, TFC and RSA results (see Figure 2). These dendrograms allow examination of the change in similarity (or closeness) of the pollen samples before and after the fermentation process. A Euclidean distance as a similarity measure was used to evaluate similarities.

The clustering results revealed four groups of pollen samples before fermentation, cutting the dendrogram tree at 0.7 of maximum distance: Italy and Spain cluster, Denmark, Netherlands, Sweden, Slovakia and Poland cluster and Lithuania, with Malta forming two separate clusters.

After spontaneous fermentation, two main distinct clusters were revealed: pollen from Lithuania and Slovakia formed one group, while the rest of the samples were more similar to each other and fell in the other group. The distance between these groups is large and expresses very distinct TPC, TFC and RSA results of these pollen samples. Interesting to note is that after spontaneous fermentation the Malta sample’s uniqueness, expressed in the first dendrogram (Figure 2a), disappeared.

The dendrogram after bacterial pollen sample fermentation showed a situation in which no clearly expressed groups were present. However, the samples were not close, except for pollen from Denmark, Italy, and Spain. It could be assumed that bacterial fermentation exposed unique properties of all the samples.

High correlation between TPC, TFC and RSA (pair-wise) was determined before and after bacterial/spontaneous fermentation (see Table 6). It is worth mentioning that all correlation coefficients have the same sign, showing the same trend (i.e., increase or decrease) for tested TPC, TFA and RSA. Neither of the tested characteristics behaved in an opposite manner.

## 4. Conclusions

Spontaneous and bacterial fermentation processes revealed a statistically significant increase of total phenolic compounds content (1.1–1.9 times), flavonoid (1.1–1.6 times) content and radical scavenging activity (1.4–2.3 times) in the tested pollen samples. The properties of the studied natural and fermented pollen depended on the geographical pollen collection area, which is related to the botanical composition of the pollen. It was determined that Lithuanian pollen had the highest total content of phenolic compounds, total flavonoids and radical scavenging activity, while pollen from Malta and Italy had the lowest values.

Lactic, ferulic, and ellagic acids, rutin, quercetin, luteolin and naringenin were identified in all samples despite the fermentation method. The study showed that the fermentation process had positive impact on pollen phenolic compound composition. In most cases, an increase in specific phenolic acids and flavonoids was observed in the range 1.2–3.1 times, and new compounds were identified after fermentation, including benzoic, vanillic and caffeic acids, and myricetin. The results and literature analysis also showed that fermentation results depend on the type of microorganisms, or even microorganisms consortia, and the botanical origin of bee-collected pollen.

Hierarchical cluster analysis allowed comparison of how close the tested pollen samples are according to measured properties. This showed that after spontaneous fermentation, the samples tended to group closer and their measured antioxidant properties became more similar, while after bacterial fermentation the results were opposite, in that all samples, except Italy, Spain and Denmark samples, became more distant with uniquely expressed antioxidant properties.

## Figures and Tables

**Table 1:** Characterization of bee-collected pollen samples.

Country	Location	GPS Coordinates	Collection Period
Lithuania	Šiauliai region, Kuršėnai	55°59′ N 22°55′ E	August 2018
Poland	Bialystok	53°08′ N 23°08′ E	July 2018
Sweden	Hagfors region	60°02′ N 13°39′ E	August 2018
Denmark	Alsgarde region	56°04′ N 12°32′ E	August 2018
Slovakia	Trnava region	48°22′ N 17°35′ E	June 2018
The Netherlands	South Holland, Gouda	52°0′ N 4°42′ E	August 2018
Republic of Malta	Northern region, Mellieha	35°57′ N 14°21′ E	August 2018
Italy	Bibbiena region	43°42′ N 11°49′ E	2018
Spain	Valencia region	39°28′ N 0°22′ W	May 2018

**Table 2:** Summary of spectrophotometric methods used for pollen samples evaluation.

Method	Total Phenolic Compounds Content	Total Flavonoid Content	Radical Scavenging Activity
Sample	8 µL of extract 8 µL Folin-Ciocalteu reagent 240 µL 4% Na_2_CO_3_	10 µL of extract 240 µL AlCl_3_ colorimetric stock solution ^1^	5.5 µL of extract 225 µL DPPH reagent
Blank	8 µL of 80% CH_3_OH 8 µL Folin-Ciocalteu reagent 240 µL 4% Na_2_CO_3_	10 µL of 80% CH_3_OH 240 µL AlCl_3_ colorimetric stock solution ^1^	5.5 µL of 80% CH_3_OH 225 µL DPPH reagent
Incubation duration	30 min	30 min	15 min
Incubation conditions	Room temperature	+4 °C	Room temperature, dark
Wavelength	694 nm	405 nm	515 nm

^1^ AlCl_3_ colorimetric stock solution consisted of 60 mL of methanol, 3 mL of 33% acetic acid, 12 mL of 5% hexamethylenetetramine, 9 mL of 10% aluminum chloride and 60 mL of bidistilled water.

**Table 3:** Calibration curve data for the reference compounds (n = 5).

Compound	Retention Time, min	Linear Regression Equation	R^2^
Lactic acid	0.30 ± 0.01	y = 1.967 × 10^3^x + 0.561 × 10^3^	0.9913
Gallic acid	0.55 ± 0.05	y = 1.779 × 10^5^x + 0.511 × 10^3^	0.9996
Benzoic acid	1.23 ± 0.02	y = 4.066 × 10^5^x − 9.802 × 10^3^	0.9992
Chlorogenic acid	2.55 ± 0.20	y = 2.586 × 10^5^x + 13.606 × 10^3^	0.9991
Vanillic acid	3.12 ± 0.02	y = 4.022 × 10^5^x + 7.491 × 10^3^	0.9988
Caffeic acid	3.41 ± 0.05	y = 2.103 × 10^5^x + 15.115 × 10^3^	0.9989
Syringic acid	3.78 ± 0.03	y = 2.552 × 10^5^x − 2.430 × 10^3^	0.9993
Salicylic acid	4.06 ± 0.10	y = 0.644 × 10^5^x + 0.347 × 10^3^	0.9996
Coumaric acid	4.34 ± 0.06	y = 2.834 × 10^5^x + 1.135 × 10^3^	0.9991
Ferulic acid	4.96 ± 0.04	y = 2.984 × 10^5^x + 3.121 × 10^3^	0.9994
Rutin	5.49 ± 0.06	y = 2.733 × 10^5^x + 5.876 × 10^3^	0.9974
Ellagic acid	5.59 ± 0.02	y = 7.343 × 10^5^x − 39.101 × 10^3^	0.9994
Coumarin	5.84 ± 0.03	y = 4.232 × 10^5^x + 0.726 × 10^3^	0.9975
Myricetin	6.82 ± 0.04	y = 2.293 × 10^5^x − 13.132 × 10^3^	0.9987
Hesperidin	7.09 ± 0.02	y = 8.740 × 10^3^x + 0.431 × 10^3^	0.9995
Quercetin	7.92 ± 0.05	y = 4.158 × 10^5^x − 15.251 × 10^3^	0.9991
Luteolin	8.07 ± 0.08	y = 2.753 × 10^5^x − 1.610 × 10^3^	0.9996
Naringenin	9.00 ± 0.04	y = 1.293 × 10^5^x − 3.407 × 10^3^	0.9982

**Table 4:** Changes of total phenolic compound content, total flavonoid content and radical scavenging activity of pollen samples before and after fermentation.

Sample	Total Phenolic Compounds Content, mg/g (RUE)			Total Flavonoid Content, mg/g (RUE)			Radical Scavenging Activity, mg/g (RUE)		
	*Before Fermentation*	Fermentation Type		*Before Fermentation*	Fermentation Type		*Before Fermentation*	Fermentation Type	
		*Bacterial*	*Spontaneous*		*Bacterial*	*Spontaneous*		*Bacterial*	*Spontaneous*
Lithuanian	11.97	22.63 *^i^*	17.99 *^i^*	6.26	9.67 *^i^*	8.85 *^i^*	9.23	14.69 *^i^*	13.62 *^i^*
Polish	10.94	16.23 *^i^*	13.75 *^i^*	4.87	7.17 *^i^*	6.05 *^i^*	6.67	11.20 *^i^*	10.24 *^i^*
Swedish	11.67	17.57 *^i^*	14.02 *^i^*	5.22	8.21 *^i^*	6.26 *^i^*	7.38	12.21 *^i^*	11.57 *^i^*
Danish	9.81	12.15 *^i^*	10.50 *^i^*	4.78	6.32 *^i^*	5.50 *^i^*	6.12	10.12 *^i^*	9.32 *^i^*
Slovak	11.29	19.98 *^i^*	18.49 *^i^*	5.36	8.24 *^i^*	7.20 *^i^*	8.08	13.04 *^i^*	11.82 *^i^*
Dutch	9.81	14.22 *^i^*	12.62 *^i^*	4.81	6.74 *^i^*	5.62 *^i^*	6.53	10.27 *^i^*	8.86 *^i^*
Maltese	8.08	10.68 *^i^*	9.28 *^i^*	3.69	5.41 *^i^*	5.00 *^i^*	2.33	5.44 *^i^*	4.98 *^i^*
Italian	8.67	12.46 *^i^*	10.34 *^i^*	4.34	6.02 *^i^*	5.16 *^i^*	4.56	8.91 *^i^*	6.17 *^i^*
Spanish	9.10	12.65 *^i^*	10.19 *^i^*	4.58	6.11 *^i^*	5.21 *^i^*	4.74	9.44 *^i^*	7.56 *^i^*
Mean	10.15	15.40	13.02	4.88	7.10	6.09	6.18	10.59	9.35
SD	1.39	4.02	3.39	0.71	1.36	1.24	2.07	2.66	2.8
Min	8.08	10.68	9.28	3.69	5.41	5.00	2.33	5.44	4.98
Max	11.97	22.63	18.49	6.26	9.67	8.85	9.23	14.69	13.62

*i*—Statistically significant change (an increase) observed after bacterial/spontaneous fermentation when *p* ≤ 0.05.

**Table 5:** Qualitative analysis results of polyphenolic compounds in natural and fermented pollen samples.

Type of Fermentation	Concentration, µg/g								
	Denmark	Sweden	Poland	Lithuania	Slovakia	Netherlands	Italy	Spain	Malta
*1*	*2*	*3*	*4*	*5*	*6*	*7*	*8*	*9*	*10*
**Gallic acid**									
Before fermentation	3.51 ± 0.13	nd	7.01 ± 0.23	31.42 ± 0.23	8.84 ± 0.50	2.31 ± 0.21	nd	2.11 ± 0.30	11.44 ± 0.22
Bacterial	4.58 ± 0.24 *^i^*	9.43 ± 0.42 *^i^*	9.93 ± 0.13 *^i^*	52.71 ± 0.41 *^i^*	22.13 ± 0.20 *^i^*	5.52 ± 0.23 *^i^*	nd	4.81 ± 0.13 *^i^*	22.71 ± 0.43 *^i^*
Spontaneous	3.33 ± 0.21	7.31 ± 0.32 *^i^*	8.14 ± 0.41 *^i^*	42.12 ± 0.42 *^i^*	14.10 ± 0.21 *^i^*	4.30 ± 0.24 *^i^*	nd	3.64 ± 0.11 *^i^*	20.13 ± 0.42 *^i^*
**Benzoic acid**									
Before fermentation	nd	nd	nd	5.51 ± 0.41	2.11 ± 0.42	1.81 ± 0.21	1.12 ± 0.22	nd	0.54 ± 0.20
Bacterial	3.12 ± 0.43 *^i^*	5.50 ± 0.22 *^i^*	3.82 ± 0.11 *^i^*	9.89 ± 0.44 *^i^*	4.93 ± 0.11 *^i^*	4.23 ± 0.23 *^i^*	1.91 ± 0.21 *^i^*	2.11 ± 0.12 *^i^*	1.50 ± 0.11 *^i^*
Spontaneous	2.41 ± 0.31 *^i^*	4.12 ± 0.41 *^i^*	2.93 ± 0.44 *^i^*	7.64 ± 0.30 *^i^*	3.34 ± 0.42 *^i^*	3.11 ± 0.21 *^i^*	1.44 ± 0.21	1.63 ± 0.21 *^i^*	1.13 ± 0.21 *^i^*
**Chlorogenic acid**									
Before fermentation	3.12 ± 0.43	3.82 ± 0.42	2.71 ± 0.40	4.22 ± 0.40	5.51 ± 0.10	3.71 ± 0.22	1.84 ± 0.11	2.50 ± 0.14	nd
Bacterial	2.01 ± 0.32 *^d^*	2.73 ± 0.34 *^d^*	2.04 ± 0.20 *^d^*	3.19 ± 0.11 *^d^*	nd *^d^*	nd *^d^*	0.93 ± 0.24 *^d^*	1.64 ± 0.12 *^d^*	nd
Spontaneous	2.53 ± 0.31 *^d^*	3.12 ± 0.41	2.42 ± 0.41	3.62 ± 0.10 *^d^*	nd *^d^*	nd *^d^*	1.44 ± 0.12 *^d^*	2.03 ± 0.11 *^d^*	nd
**Vanillic acid**									
Before fermentation	nd	nd	nd	nd	nd	nd	1.14 ± 0.31	nd	nd
Bacterial	2.42 ± 0.41 *^i^*	46.60 ± 0.61 *^i^*	58.72 ± 0.21 *^i^*	68.90 ± 0.51 *^i^*	43.32 ± 0.51 *^i^*	34.10 ± 0.20 *^i^*	22.38 ± 0.54 *^i^*	23.42 ± 0.12 *^i^*	21.64 ± 0.43 *^i^*
Spontaneous	1.91 ± 0.42 *^i^*	31.63 ± 0.41 *^i^*	40.22 ± 0.54 *^i^*	45.83 ± 0.12 *^i^*	32.81 ± 0.62 *^i^*	22.24 ± 0.51 *^i^*	15.13 ± 0.40 *^i^*	13.43 ± 0.41 *^i^*	12.61 ± 0.21 *^i^*
**Caffeic acid**									
Before fermentation	2.32 ± 0.23	nd	2.14 ± 0.11	nd	nd	1.92 ± 0.21	2.53 ± 0.10	nd	2.12 ± 0.31
Bacterial	5.49 ± 0.21 *^i^*	nd	5.70 ± 0.41 *^i^*	8.14 ± 0.23 *^i^*	nd	4.91 ± 0.12 *^i^*	4.24 ± 0.21 *^i^*	nd	4.84 ± 0.13 *^i^*
Spontaneous	4.34 ± 0.24 *^i^*	nd	4.42 ± 0.30 *^i^*	6.33 ± 0.31 *^i^*	nd	3.83 ± 0.44 *^i^*	3.14 ± 0.23 *^i^*	nd	3.63 ± 0.12 *^i^*
**Syringic acid**									
Before fermentation	3.51 ± 0.11	nd	7.04 ± 0.20	8.81 ± 0.51	6.63 ± 0.33	3.31 ± 0.22	1.92 ± 0.21	2.14 ± 0.33	1.54 ± 0.20
Bacterial	4.62 ± 0.24 *^i^*	9.41 ± 0.41 *^i^*	nd *^d^*	22.11 ± 0.22 *^i^*	13.44 ± 0.42 *^i^*	5.49 ± 0.20 *^i^*	3.13 ± 0.12 *^i^*	4.13 ± 0.11 *^i^*	1.91 ± 0.14 *^i^*
Spontaneous	3.33 ± 0.21	7.32 ± 0.31 *^i^*	nd *^d^*	14.13 ± 0.24 *^i^*	9.42 ± 0.31 *^i^*	4.32 ± 0.21 *^i^*	2.24 ± 0.11	3.02 ± 0.13 *^i^*	1.70 ± 0.11
**Salicylic acid**									
Before fermentation	10.39 ± 0.31	14.51 ± 0.61	13.67 ± 0.37	17.71 ± 0.37	16.61 ± 0.40	nd	nd	7.11 ± 0.20	6.71 ± 0.31
Bacterial	17.59 ± 0.44 *^i^*	21.30 ± 0.43 *^i^*	18.76 ± 0.39 *^i^*	23.31 ± 0.49 *^i^*	22.10 ± 0.51 *^i^*	nd	nd	14.09 ± 0.21 *^i^*	10.39 ± 0.46 *^i^*
Spontaneous	11.91 ± 0.10 *^i^*	19.61 ± 0.12 *^i^*	16.21 ± 0.20 *^i^*	20.16 ± 0.19 *^i^*	19.83 ± 0.55 *^i^*	nd	nd	12.21 ± 0.45 *^i^*	9.45 ± 0.41 *^i^*
**Coumaric acid**									
Before fermentation	1.31 ± 0.22	nd	nd	3.61 ± 0.24	1.91 ± 0.21	1.04 ± 0.30	0.31 ± 0.11	0.63 ± 0.21	nd
Bacterial	2.22 ± 0.13 *^i^*	nd	nd	5.63 ± 0.21 *^i^*	3.71 ± 0.21 *^i^*	2.44 ± 0.21 *^i^*	1.54 ± 0.21 *^i^*	2.44 ± 0.64 *^i^*	nd
Spontaneous	1.61 ± 0.20	nd	nd	4.24 ± 0.20 *^i^*	2.92 ± 0.30 *^i^*	1.64 ± 0.30	1.14 ± 0.10 *^i^*	1.30 ± 0.41 *^i^*	nd
**Ferulic acid**									
Before fermentation	13.12 ± 0.23	26.61 ± 0.63	23.10 ± 0.44	38.14 ± 0.54	27.71 ± 0.42	13.42 ± 0.33	9.10 ± 0.34	9.42 ± 0.32	7.91 ± 0.11
Bacterial	31.31 ± 0.41 *^i^*	46.64 ± 0.61 *^i^*	43.32 ± 0.52 *^i^*	68.90 ± 0.51 *^i^*	58.73 ± 0.24 *^i^*	34.13 ± 0.21 *^i^*	22.43 ± 0.54 *^i^*	23.43 ± 0.13 *^i^*	21.63 ± 0.44 *^i^*
Spontaneous	21.94 ± 0.40 *^i^*	31.62 ± 0.41 *^i^*	32.84 ± 0.64 *^i^*	45.83 ± 0.10 *^i^*	40.22 ± 0.51 *^i^*	22.24 ± 0.50 *^i^*	15.12 ± 0.41 *^i^*	13.44 ± 0.41 *^i^*	12.62 ± 0.20 *^i^*
**Coumarin**									
Before fermentation	7.11 ± 0.22	8.92 ± 0.21	nd	nd	nd	nd	6.72 ± 0.33	nd	nd
Bacterial	21.32 ± 0.42 *^i^*	26.63 ± 0.62 *^i^*	nd	nd	nd	nd	10.41 ± 0.54 *^i^*	nd	nd
Spontaneous	11.91 ± 0.10 *^i^*	21.61 ± 0.13 *^i^*	nd	nd	nd	nd	9.52 ± 0.41 *^i^*	nd	nd
**Ellagic acid**									
Before fermentation	11.12 ± 0.22	11.41 ± 0.63	17.72 ± 0.43	28.12 ± 0.22	19.91 ± 0.10	10.43 ± 0.34	3.41 ± 0.11	6.61 ± 0.33	2.71 ± 0.13
Bacterial	19.31 ± 0.44 *^i^*	23.33 ± 0.51 *^i^*	46.71 ± 0.22 *^i^*	58.91 ± 0.42 *^i^*	36.62 ± 0.62 *^i^*	14.12 ± 0.21 *^i^*	7.52 ± 0.23 *^i^*	9.43 ± 0.14 *^i^*	5.62 ± 0.24 *^i^*
Spontaneous	18.43 ± 0.12 *^i^*	12.82 ± 0.62 *^i^*	30.22 ± 0.21 *^i^*	35.82 ± 0.11 *^i^*	21.63 ± 0.11 *^i^*	12.21 ± 0.50 *^i^*	6.64 ± 0.21 *^i^*	8.34 ± 0.42 *^i^*	3.90 ± 0.21 *^i^*
**Rutin**									
Before fermentation	31.12 ± 0.20	41.61 ± 0.31	41.70 ± 0.43	54.12 ± 0.52	48.13 ± 0.43	33.12 ± 0.30	27.61 ± 0.33	27.40 ± 0.33	21.40 ± 0.21
Bacterial	52.34 ± 0.42 *^i^*	64.64 ± 0.63 *^i^*	75.74 ± 0.21 *^i^*	89.93 ± 0.53 *^i^*	63.32 ± 0.54 *^i^*	57.73 ± 0.22 *^i^*	44.63 ± 0.54 *^i^*	41.21 ± 0.14 *^i^*	40.04 ± 0.41 *^i^*
Spontaneous	42.93 ± 0.41 *^i^*	56.63 ± 0.42 *^i^*	61.23 ± 0.52 *^i^*	64.84 ± 0.12 *^i^*	52.22 ± 0.61 *^i^*	45.10 ± 0.51 *^i^*	37.14 ± 0.42 *^i^*	33.43 ± 0.42 *^i^*	30.63 ± 0.21 *^i^*
**Myricetin**									
Before fermentation	6.52 ± 0.14	nd	nd	5.69 ± 0.09	6.51 ± 0.10	4.86 ± 0.21	6.49 ± 0.33	1.50 ± 0.41	4.44 ± 0.50
Bacterial	16.29 ± 0.43 *^i^*	11.59 ± 0.33 *^i^*	18.71 ± 0.43 *^i^*	17.10 ± 0.21 *^i^*	18.12 ± 0.43 *^i^*	8.41 ± 0.16 *^i^*	8.91 ± 0.41 *^i^*	6.23 ± 0.09 *^i^*	11.61 ± 0.41 *^i^*
Spontaneous	14.89 ± 0.22 *^i^*	8.91 ± 0.40 *^i^*	10.91 ± 0.10 *^i^*	16.01 ± 0.19 *^i^*	7.68 ± 0.21 *^i^*	8.03 ± 0.20 *^i^*	7.43 ± 0.86 *^i^*	4.34 ± 0.20 *^i^*	10.89 ± 0.12 *^i^*
**Hesperidin**									
Before fermentation	nd	nd	nd	11.55 ± 0.21	nd	5.41 ± 0.21	nd	nd	2.12 ± 0.19
Bacterial	19.09 ± 0.35 *^i^*	nd	nd	26.51 ± 0.18 *^i^*	nd	10.20 ± 0.35 *^i^*	nd	9.93 ± 0.13 *^i^*	6.64 ± 0.21 *^i^*
Spontaneous	9.11 ± 0.19 *^i^*	nd	nd	19.39 ± 0.19 *^i^*	nd	9.12 ± 0.29 *^i^*	nd	6.88 ± 0.11 *^i^*	3.86 ± 0.45 *^i^*
**Quercetin**									
Before fermentation	22.72 ± 0.76	40.21 ± 0.77	36.31 ± 0.19	58.10 ± 0.48	47.71 ± 0.38	7.11 ± 0.12	9.03 ± 0.28	4.10 ± 0.41	6.62 ± 0.41
Bacterial	40.29 ± 0.51 *^i^*	61.09 ± 0.45 *^i^*	49.12 ± 0.29 *^i^*	78.86 ± 0.46 *^i^*	68.69 ± 0.19 *^i^*	21.40 ± 0.11 *^i^*	20.41 ± 0.46 *^i^*	8.13 ± 0.24 *^i^*	20.59 ± 0.36 *^i^*
Spontaneous	30.81 ± 0.55 *^i^*	45.88 ± 0.10 *^i^*	44.43 ± 0.22 *^i^*	65.81 ± 0.11 *^i^*	60.21 ± 0.12 *^i^*	10.41 ± 0.35 *^i^*	11.10 ± 0.39 *^i^*	7.31 ± 0.29 *^i^*	9.57 ± 0.19 *^i^*
**Luteolin**									
Before fermentation	33.10 ± 0.24	43.61 ± 0.26	43.12 ± 0.40	58.13 ± 0.49	47.68 ± 0.37	33.41 ± 0.30	nd	29.41 ± 0.27	29.61 ± 0.22
Bacterial	51.29 ± 0.40 *^i^*	66.59 ± 0.61 *^i^*	63.33 ± 0.51 *^i^*	88.90 ± 0.46 *^i^*	78.71 ± 0.22 *^i^*	54.12 ± 0.21 *^i^*	42.42 ± 0.51 *^i^*	43.36 ± 0.10 *^i^*	41.56 ± 0.35 *^i^*
Spontaneous	41.90 ± 0.35 *^i^*	51.60 ± 0.39 *^i^*	52.79 ± 0.55 *^i^*	65.81 ± 0.09 *^i^*	60.21 ± 0.54 *^i^*	42.23 ± 0.46 *^i^*	35.14 ± 0.42 *^i^*	33.39 ± 0.35 *^i^*	32.62 ± 0.21 *^i^*
**Naringenin**									
Before fermentation	27.61 ± 0.29	31.59 ± 0.30	41.69 ± 0.39	59.21 ± 0.51	49.69 ± 0.36	21.61 ± 0.29	34.41 ± 0.26	27.39 ± 0.30	21.09 ± 0.21
Bacterial	44.62 ± 0.51 *^i^*	54.57 ± 0.60 *^i^*	91.81 ± 0.21 *^i^*	135.03 ± 0.49 *^i^*	82.21 ± 0.49 *^i^*	31.71 ± 0.56 *^i^*	79.10 ± 0.19 *^i^*	42.32 ± 0.36 *^i^*	41.22 ± 0.09 *^i^*
Spontaneous	37.13 ± 0.35 *^i^*	46.61 ± 0.35 *^i^*	67.82 ± 0.45 *^i^*	124.81 ± 0.09 *^i^*	74.13 ± 0.55 *^i^*	28.71 ± 0.34 *^i^*	45.13 ± 0.46 *^i^*	33.41 ± 0.39 *^i^*	32.91 ± 0.36 *^i^*

nd—not detected. *i*, *d*—statistically significant changes (increase or decrease, respectively) observed after bacterial/spontaneous fermentation, when *p* ≤ 0.05.

**Table 6:** Correlation coefficients between total phenolic compounds content (TPC), total flavonoid content (TFC), and radical scavenging activity (RSA).

Criteria	Before Fermentation			Bacterial Fermentation			Spontaneous Fermentation		
	TPC	TFC	RSA	TPC	TFC	RSA	TPC	TFC	RSA
**TPC**	1	0.905	0.945	1	0.979	0.924	1	0.906	0.892
**TFC**		1	0.956		1	0.935		1	0.872
**RSA**			1			1			1

## Data Availability

Data is contained within the article and supplementary material.

## Supplementary Materials

The following supporting information can be downloaded at: https://www.mdpi.com/article/10.3390/antiox11040645/s1, Figure S1: Visual appearance of the samples.
