# Supplementary material for: The Impact of Fermentation on Bee Pollen Polyphenolic Compounds Composition
Source: Antioxidants (Basel). 2022 Mar 28;11(4):645. doi: 10.3390/antiox11040645 (PMC9032161; doi:10.3390/antiox11040645)
Supplement: Supplementary file 1 [file antioxidants-11-00645-s001.zip › antioxidants-1639480-supplementary.pdf]

Supplementary Material

The Impact of Fermentation on Bee Pollen Polyphenolic Compounds Composition

Vaida Adaškevičiūtė, Vilma Kaškonienė, Karolina Barčauskaitė, Paulius Kaškonas and Audrius Maruška

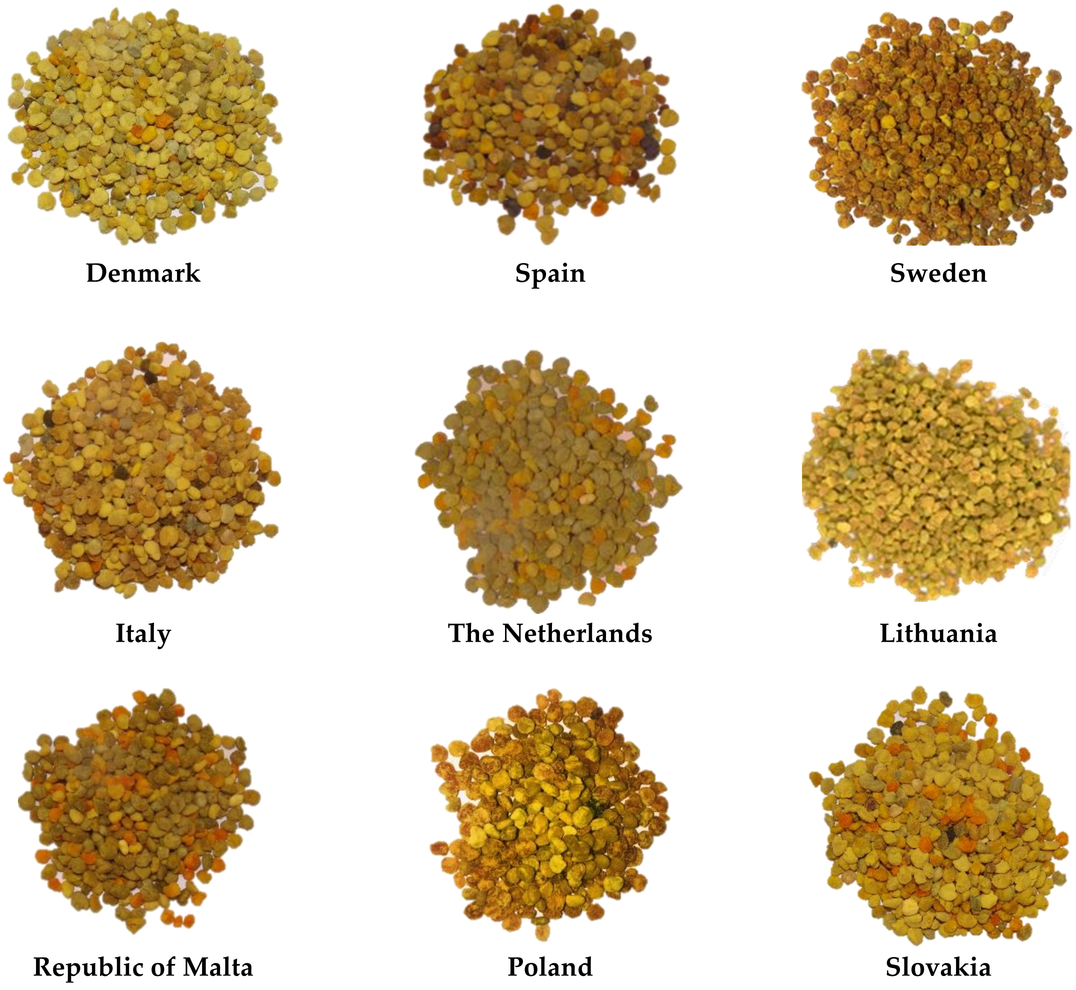

Figure S1. Visual appearance of the samples
